# Optimizing the trajectory of deep brain stimulation leads reduces RF heating during MRI at 3 T: Characteristics and clinical translation

**DOI:** 10.1109/EMBC40787.2023.10340979

**Published:** 2023-07

**Authors:** Jasmine Vu, Bhumi Bhusal, Joshua Rosenow, Julie Pilitsis, Laleh Golestanirad

**Affiliations:** Department of Biomedical Engineering, McCormick School of Engineering, Northwestern University, Evanston, IL, USA and the Department of Radiology, Feinberg School of Medicine, Northwestern University, Chicago, IL, USA; Department of Radiology, Feinberg School of Medicine, Northwestern University, Chicago, IL, USA; Department of Neurosurgery, Feinberg School of Medicine, Northwestern University, Chicago, IL, USA; Department of Neurosciences & Experimental Therapeutics, Albany Medical College, Albany, NY, USA; Department of Biomedical Engineering, McCormick School of Engineering, Northwestern University, Evanston, IL, USA and the Department of Radiology, Feinberg School of Medicine, Northwestern University, Chicago, IL, USA

## Abstract

Radiofrequency (RF) induced tissue heating around deep brain stimulation (DBS) leads is a well-known safety risk during magnetic resonance imaging (MRI), hindering routine protocols for patients. Known factors that contribute to variations in the magnitude of RF heating across patients include the implanted lead’s trajectory and its orientation with respect to the MRI electric fields. Currently, there are no consistent requirements for surgically implanting the extracranial portion of the DBS lead. Recent studies have shown that incorporating concentric loops in the extracranial trajectory of the lead can reduce RF heating, but the optimal positioning of the loop is unknown. In this study, we evaluated RF heating of 77 unique lead trajectories to determine how different characteristics of the trajectory affect RF heating during MRI at 3 T. We performed phantom experiments with commercial DBS systems from two manufacturers to determine how consistently modifying the lead trajectory mitigates RF heating. We also presented the first surgical implementation of these modified trajectories in patients. Low-heating trajectories included small concentric loops near the surgical burr hole which were readily implemented during the surgical procedure; these trajectories generated nearly a 2-fold reduction in RF heating compared to unmodified trajectories.

## I. Introduction

Deep brain stimulation (DBS) is a neurosurgical procedure where electrical stimulation is delivered to specific subcortical targets in the brain, providing therapeutic benefits to patients with movement disorders and other neurological diseases [1]–[4]. For patients with implanted DBS systems, magnetic resonance imaging (MRI) is highly useful, allowing for postoperative monitoring, target verification, and localization of the electrodes [5], [6], along with elucidating the functional effects of stimulation on affected brain networks [7]. As the field of DBS therapy advances, the need for application of higher field strength MRI (i.e., 3 T and above) for improved contrast-to-noise ratio is increasingly imperative [8], [9].

Radiofrequency (RF) induced heating is a well-established safety concern, limiting the types of MRI exams patients with DBS devices can receive. The implanted DBS lead behaves like an antenna when coupled with the electric field of the MRI transmit coil, which increases the specific absorption rate (SAR) of RF energy deposited in the tissue surrounding the lead’s tip [10]–[12]. To mitigate the safety concerns associated with RF heating, DBS manufacturers have established stringent device-specific guidelines for MRI protocols. Specifically, most neuroimaging procedures are performed in a horizontal closed-bore scanner at 1.5 T. Pulse sequences should also adhere to heating-related thresholds, (e.g., B_1_^+^_rms_ < 1.1 μT or whole-head SAR < 0.1 W/kg—30 times below the FDA’s limit for scanning in the absence of implants [13], [14]). Such guidelines limit clinical MRI for patients with DBS devices.

One proposed technique for reducing RF heating is to modify the extracranial portion of the DBS lead trajectory during surgery [15]. Previous work showed that manipulating the lead trajectory reduced RF heating by 3-folds compared to unmodified trajectories [16]. Surgical guidelines have been established for implanting the intracranial trajectory of the lead, specifying the necessary entry point on the skull and the angle of insertion to target the intended brain structure. However, the extracranial portion of the lead does not contribute to the therapeutic effects of DBS for patients, resulting in a lack of trajectory implantation guidelines. Substantial variations in the extracranial lead trajectories across patients have been found, which in turn leads to highly variable—and unpredictable—RF tissue heating [17]. One proposed modified trajectory configuration involves surgically shaping the extracranial portion of the DBS lead into concentric loops near the surgical burr-hole [17], [18]. However, the optimal positioning and topology of the loops remain unknown. Prior *in vitro* studies have implemented nondescript loops near the surgical burr-hole, but the limited configurations did not fully consider all potential loop dimensions and trajectory positions in the phantom [19]–[21].

In this study, we performed the first large-scale study to demonstrate the effectiveness of modifying the extracranial DBS lead trajectory to minimize RF heating during MRI at 3 T. We compared the RF heating of 77 unique lead trajectories across two commercial DBS systems.

## II. Methods

### A. Lead Trajectory Parameters

**Figure 1.**
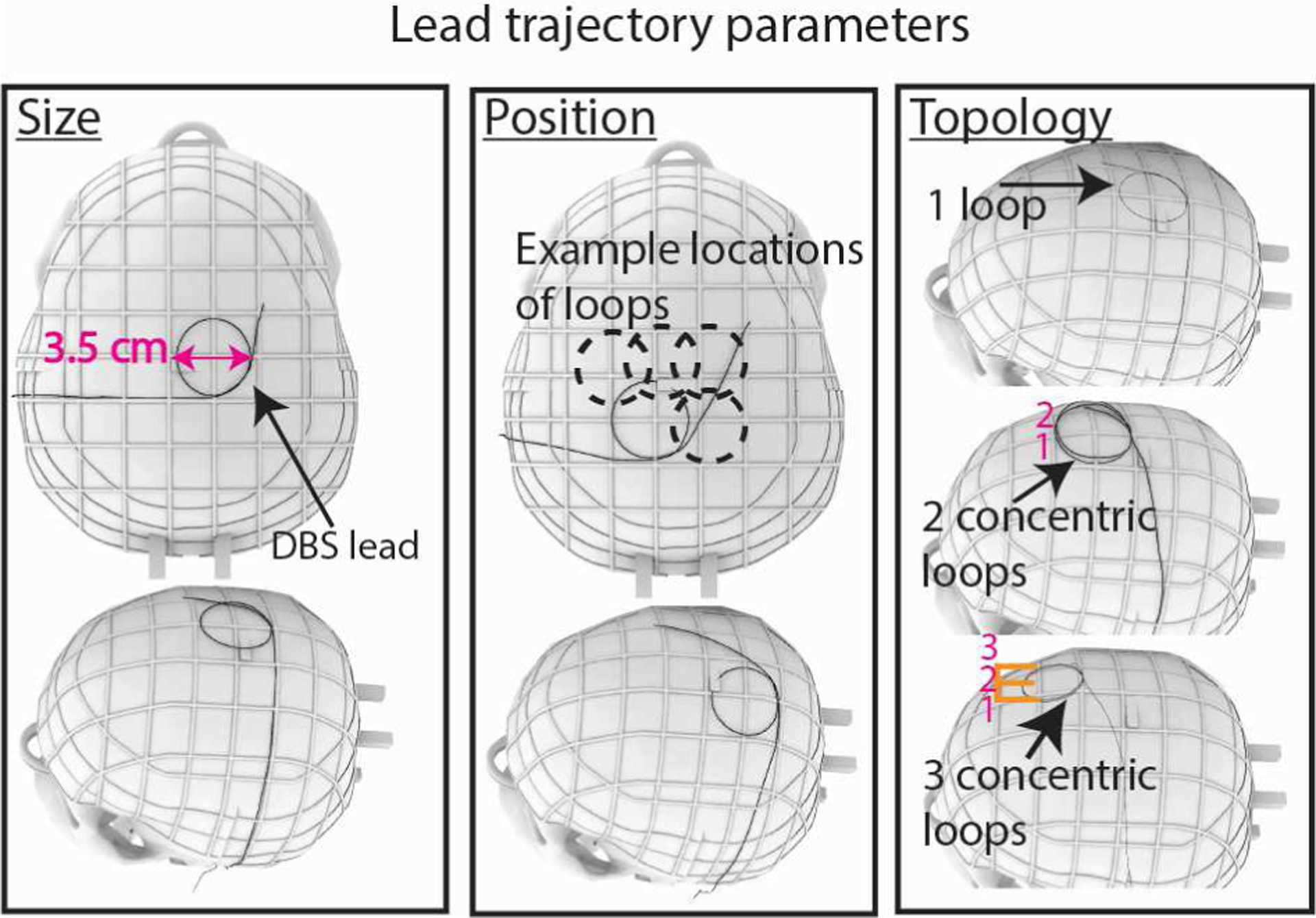
Extracranial DBS lead trajectory parameters evaluated include the diameter of the loops, the position of the loops on the skull, and the topology (i.e., the number of concentric loops).

**Figure 2.**
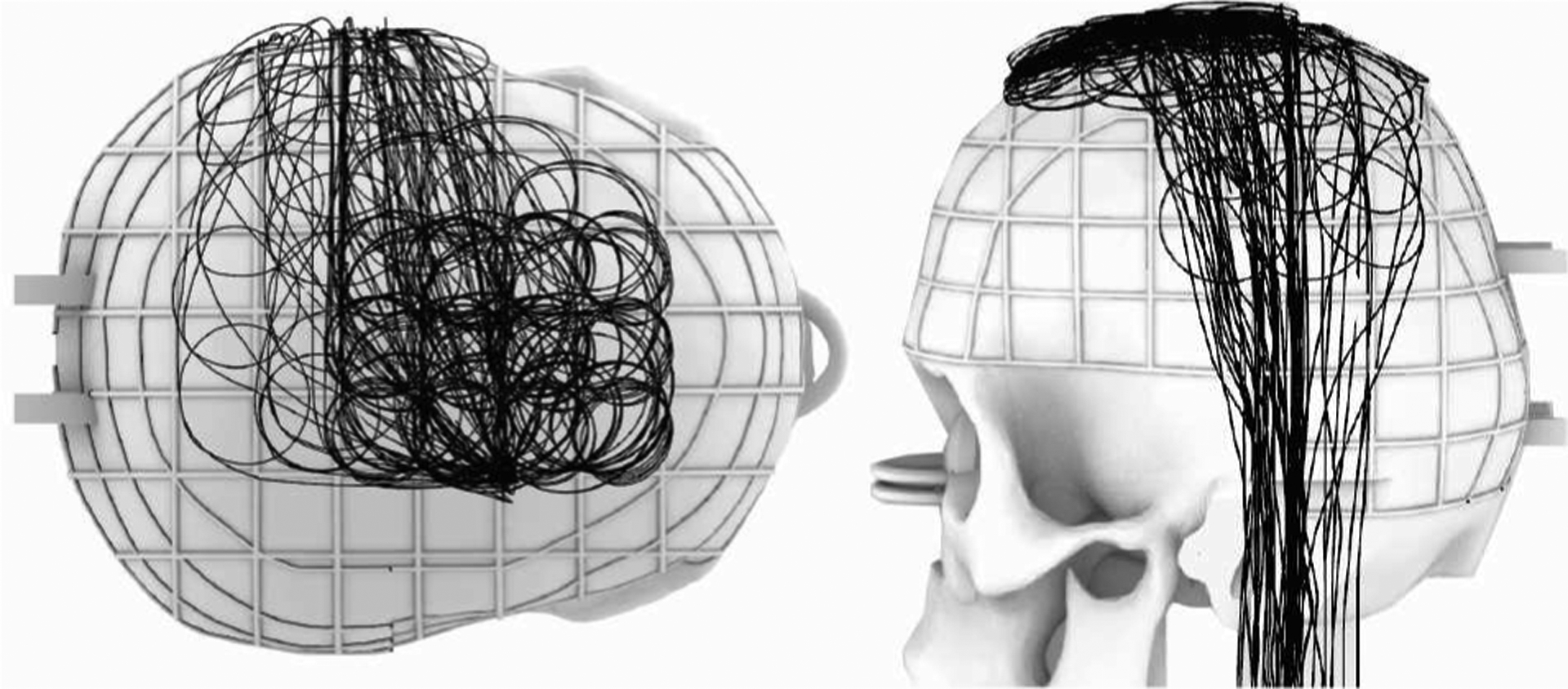
Superposition of all the trajectories evaluated in this study for a total of 77 unique trajectories.

We assessed how different parameters of the extracranial lead trajectory affect RF heating including the number of concentric loops (1–3 loops), the diameter of the concentric loops (2.5–4.5 cm with 0.5 cm increments), and the position of the loops on the skull (Fig. 1). Trajectory characteristics were selected based on the literature and retrospective analysis of imaging data from patients undergoing DBS surgery at our institutions to determine surgically and anatomically feasible trajectories [19]–[21]. All loops were coiled in a clockwise direction. Additionally, we developed a process for precise replication of the intended trajectories during the RF heating experiments which ensured that the experiments were reproducible across commercial DBS leads. For this, we first created 3D models of the proposed lead trajectories in a CAD tool (Rhino 7.0, Robert McNeel & Associates, Seattle, WA). We then translated the trajectories to the commercial DBS systems using 3D printed trajectory guides, similar to the method described in [22]. A total of 77 unique trajectories were evaluated for both DBS systems (Fig. 2). All experimental configurations were cases of unilateral DBS systems (i.e., only one DBS lead connected to the IPG).

### B. DBS Devices and Experimental Setup

**Figure 3.**
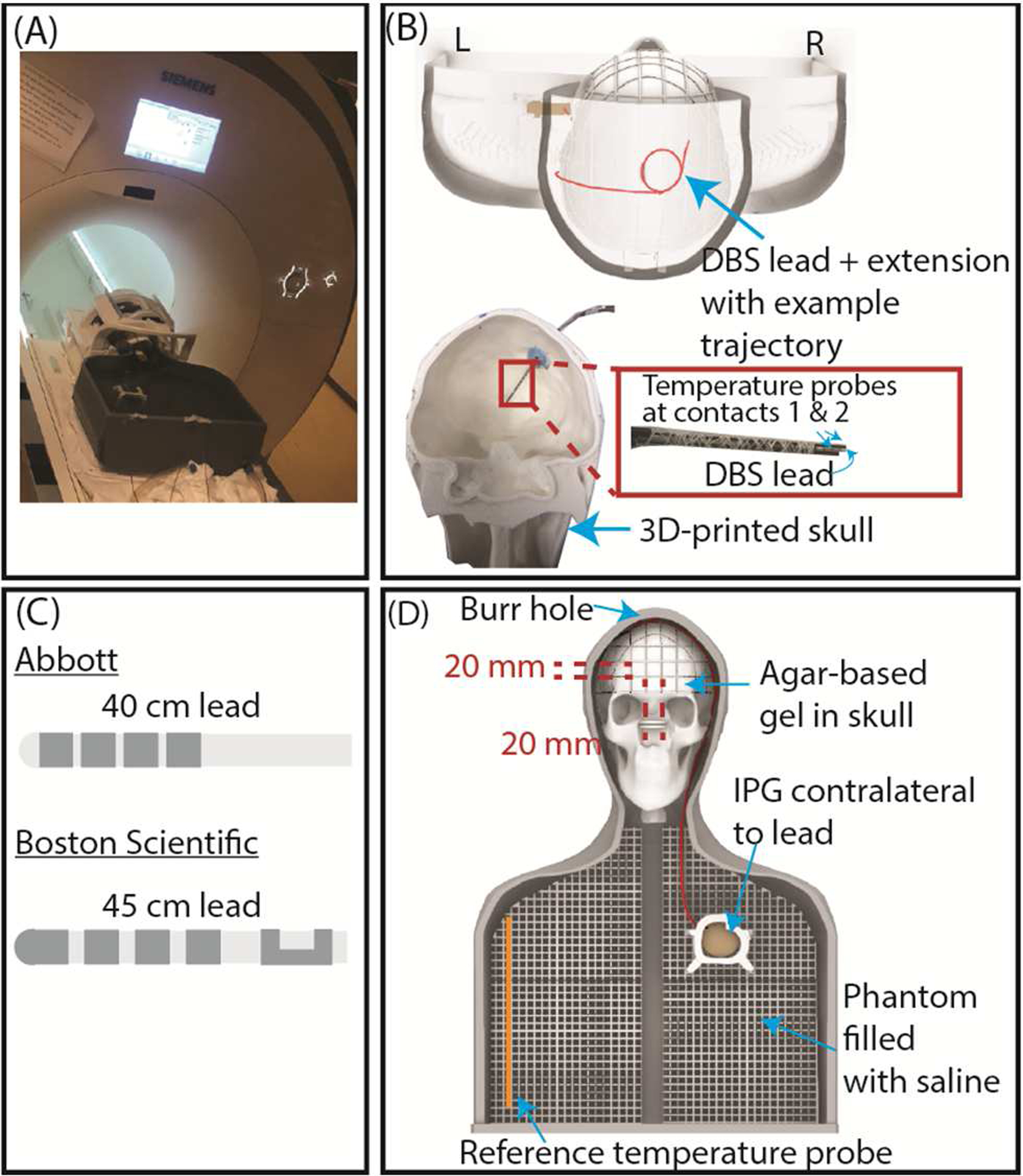
(A) Experimental setup at a 3 T Siemens Prisma scanner. (B) 3D rendering of the 3D-printed phantom with an example lead trajectory. The DBS lead and temperature probes were inserted into the skull. (C) Simplified renderings of the distal end of the DBS leads. (D) Phantom setup with an implanted full DBS system.

Experiments were conducted with full DBS systems from Abbott (40 cm lead model 6172, 50 cm extension model 6371, and Infinity-5 IPG) and Boston Scientific (45 cm lead model DB-2202–45, 55 cm extension model NM-3138–55, and Vercise Gevia IPG) (Fig. 3). Both DBS leads were individually implanted in the right hemisphere (i.e., entry point on the right side of the respective skull) with the target and angle of insertion mimicking the subthalamic nucleus. The extension connecting the lead to the IPG was situated laterally along the neck with any excess length looped around the IPG, and the IPG was placed in the left pectoral region representing a contralateral lead-IPG configuration.

The phantom consisted of a skull filled with an agar-based gel (σ = 0.47 S/m, *ε*_r_ = 78) prepared by mixing 32 g/L of edible agar (Landor Trading Company, gel strength = 900 g/cm^2^), 5 g/L of sodium benzoate (Sigma Aldrich), and saline solution (1.55 gNaCl/L), and the head-torso shell filled with 18 L of saline solution (σ = 0.50 S/m, *ε*_r_ = 80) mimicking the conductivity of average human tissue. Details about the fabrication of the anthropomorphic phantom are provided elsewhere [23]. Fiber-optic temperature probes (Osensa, Burnaby, BC, Canada, resolution = 0.01 °C) were attached to the distal end of the leads to measure the temperature increase, ΔT_max_, in the gel surrounding the lead-tip. The temperature was measured throughout the RF exposure with ample cooling time between configurations.

### C. RF Heating Experiments

Experiments were performed in a 3 T Siemens Prisma MRI scanner with the body transmit coil and 20-channel receive head coil (Siemens Healthineers, Erlangen, Germany). RF exposure was generated with a high-SAR pulse sequence (T1-weighted turbo spin echo dark fluid, TR = 2750 ms, TE = 8.2 ms, FA = 170°, acquisition time = 381 seconds, B_1_^+^rms = 2.7 μT, axial slices). All experiments were performed with the phantom in the head-first, supine position, and a brain imaging landmark such that the eyebrows/tip of the DBS lead was at the scanner’s isocenter.

### D. Application of Modified Lead Trajectories in Patients

**Figure 4.**
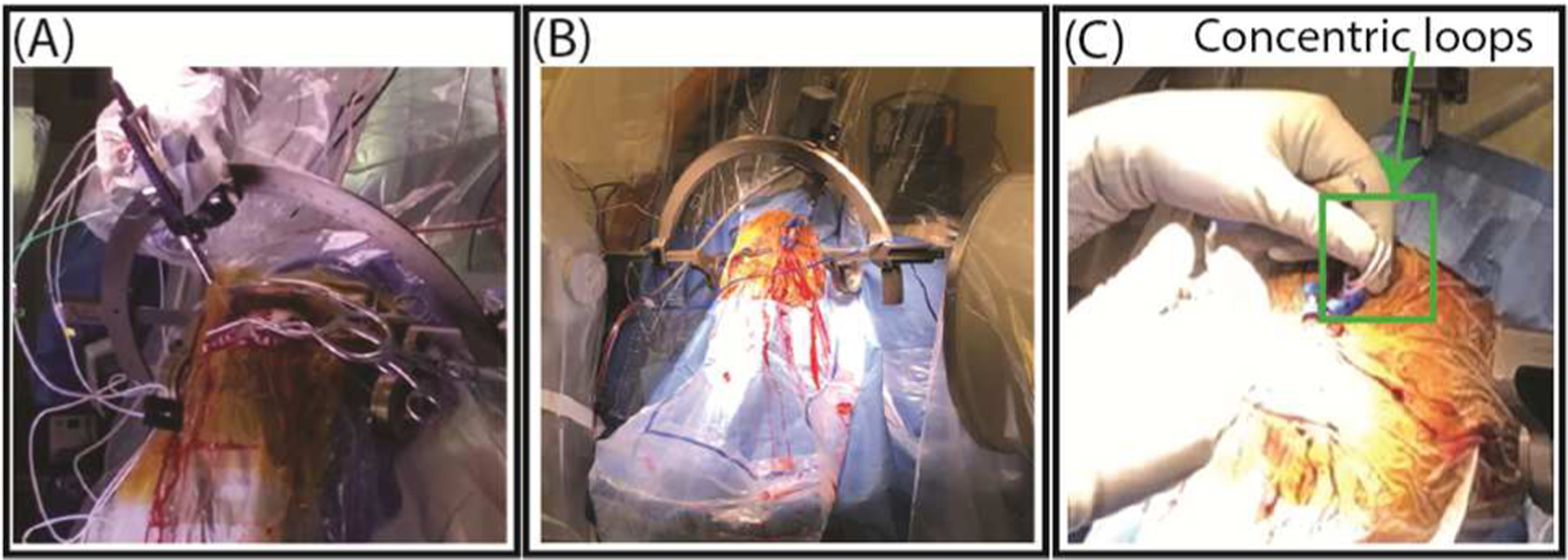
DBS implantation surgery at (A) Albany Medical Center and (B) Northwestern Memorial Hospital. (C) Concentric loops implemented near the surgical burr hole based on results from the phantom experiments.

To determine the effectiveness of surgically modifying the extracranial lead trajectory, two neurosurgeons (J. R. at Northwestern Memorial Hospital and J. P. at Albany Medical Center) were instructed to implement characteristics of low-heating trajectories (i.e., 2–3 concentric, overlapping loops near the surgical burr hole) (Fig. 4). The concentric loops were coiled and inserted beneath the scalp posterior to the burr hole.

**Figure 5.**
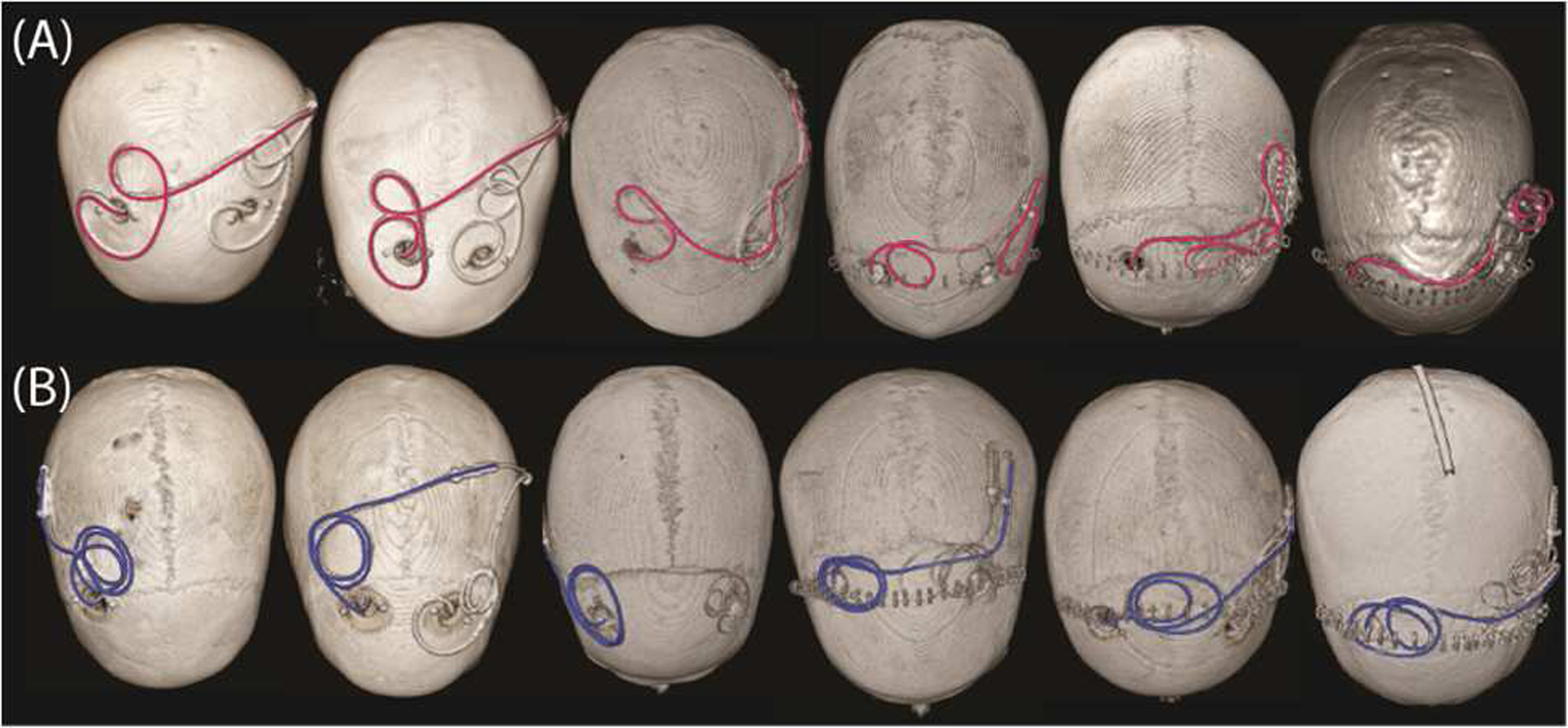
3D surface rendered views of CT images of patients with (A) unmodified (highlighted in magenta) and (B) modified (highlighted in blue) DBS lead trajectories that were replicated during phantom experiments.

Following DBS surgery, phantom experiments were performed with six recently implemented low-heating lead trajectories and six lead trajectories from the same neurosurgeons without modifying the trajectory to compare the effectiveness of surgical lead modification (Fig. 5). To replicate the clinical trajectories during the RF heating experiments, lead trajectories were segmented from postoperative computed tomography (CT) images using 3D slicer 5.3.0 (http://slicer.org), processed in the Rhino CAD tool, and 3D-printed. The trajectories were replicated with the Boston Scientific DBS system during the experiments. Retrospective use of the patients’ imaging data for the purpose of modeling was approved by Northwestern Memorial Hospital and Albany Medical Center’s institutional review boards.

## III. Results

### A. RF Heating Measurements

**Figure 6.**
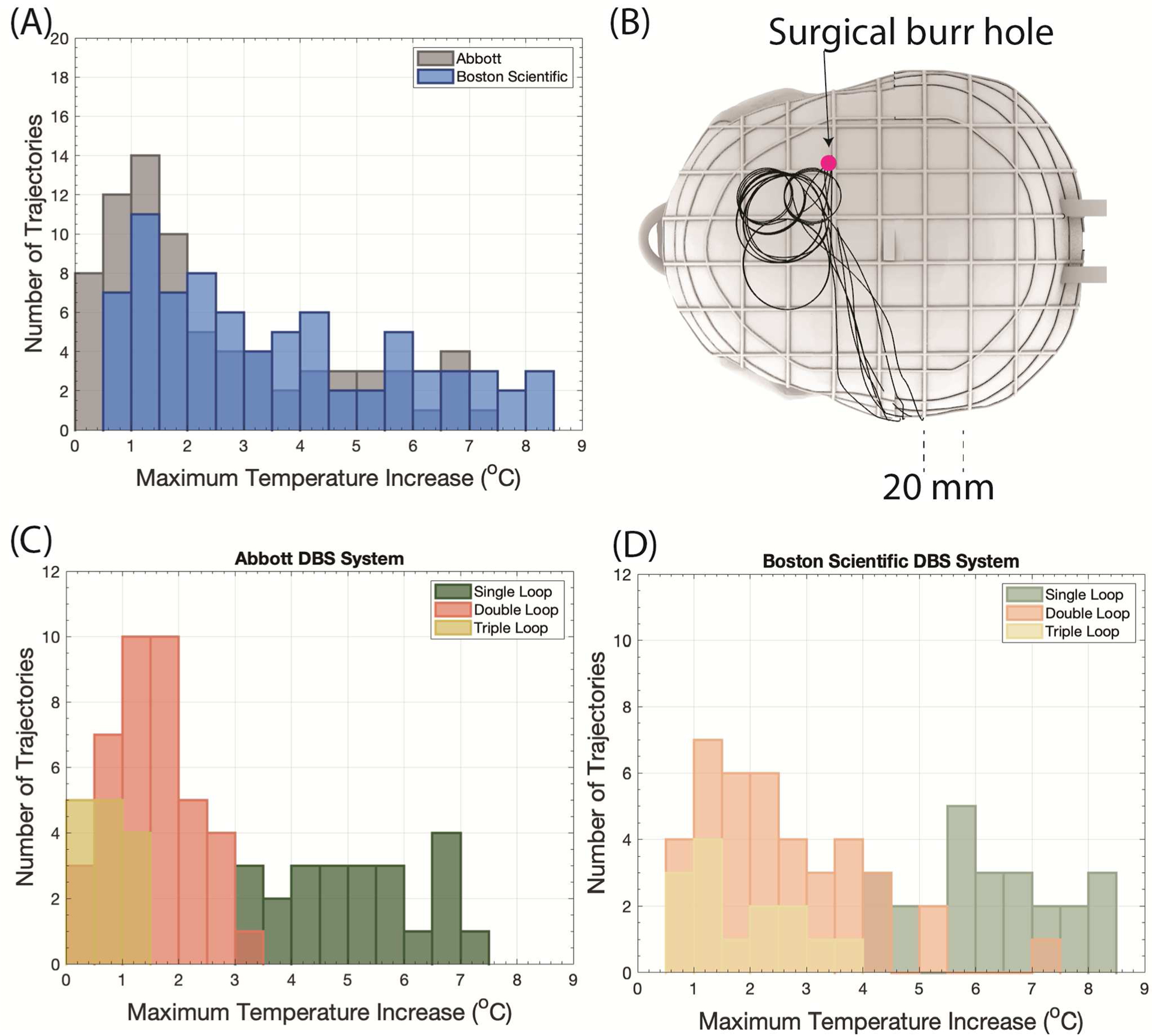
(A) Distribution of ΔT_max_ for all lead trajectories froom phantom experiments performed with the Abbott and Boston Scientific DBS systems. (B) Superposition of low heating trajectories where ΔT_max_ < °C, illustrating concentric loops near the surgical burr hole. Distribution of ΔT_max_ categorized by the number of concentric loops for the (C) Abbott DBS system and the (D) Boston Scientific DBS system.

Across the 77 lead trajectories, the mean ± standard deviation of ΔT_max_ was 2.47 ± 1.98 °C with a range of 0.24–7.34 °C for the Abbott DBS system. The mean ± standard deviation of ΔT_max_ was 3.57 ± 2.22 °C with a range of 0.58–8.28 °C for the Boston Scientific DBS system. Furthermore, as the number of concentric loops increased, ΔT_max_ generally decreased (Fig. 6).

For the single loop topology, the mean ± standard deviation of ΔT_max_ was 5.16 ± 1.18 °C and 6.28 ± 1.24 °C for the Abbott and Boston Scientific DBS systems, respectively. Similarly, double loop configurations resulted in a mean ± standard deviation of ΔT_max_ of 1.54 ± 0.76 °C and 2.60 ± 1.43°C for the Abbott and Boston Scientific DBS systems, respectively. Triple loop configurations generated a mean ± standard deviation of ΔT_max_ of 0.73 ± 0.40 °C and 1.88 ± 1.05 °C for the Abbott and Boston Scientific DBS systems, respectively.

The position of the concentric loops had a nontrivial effect on the magnitude of RF heating; concentric loops of low-heating trajectories were located within 40 mm (radially) of the surgical burr hole.

For both Abbott and Boston Scientific systems, low-heating trajectories had 2–3 small concentric loops near the surgical burr hole.

### B. Reduced RF Heating of Patient-derived Modified Trajectories

**Figure 7.**
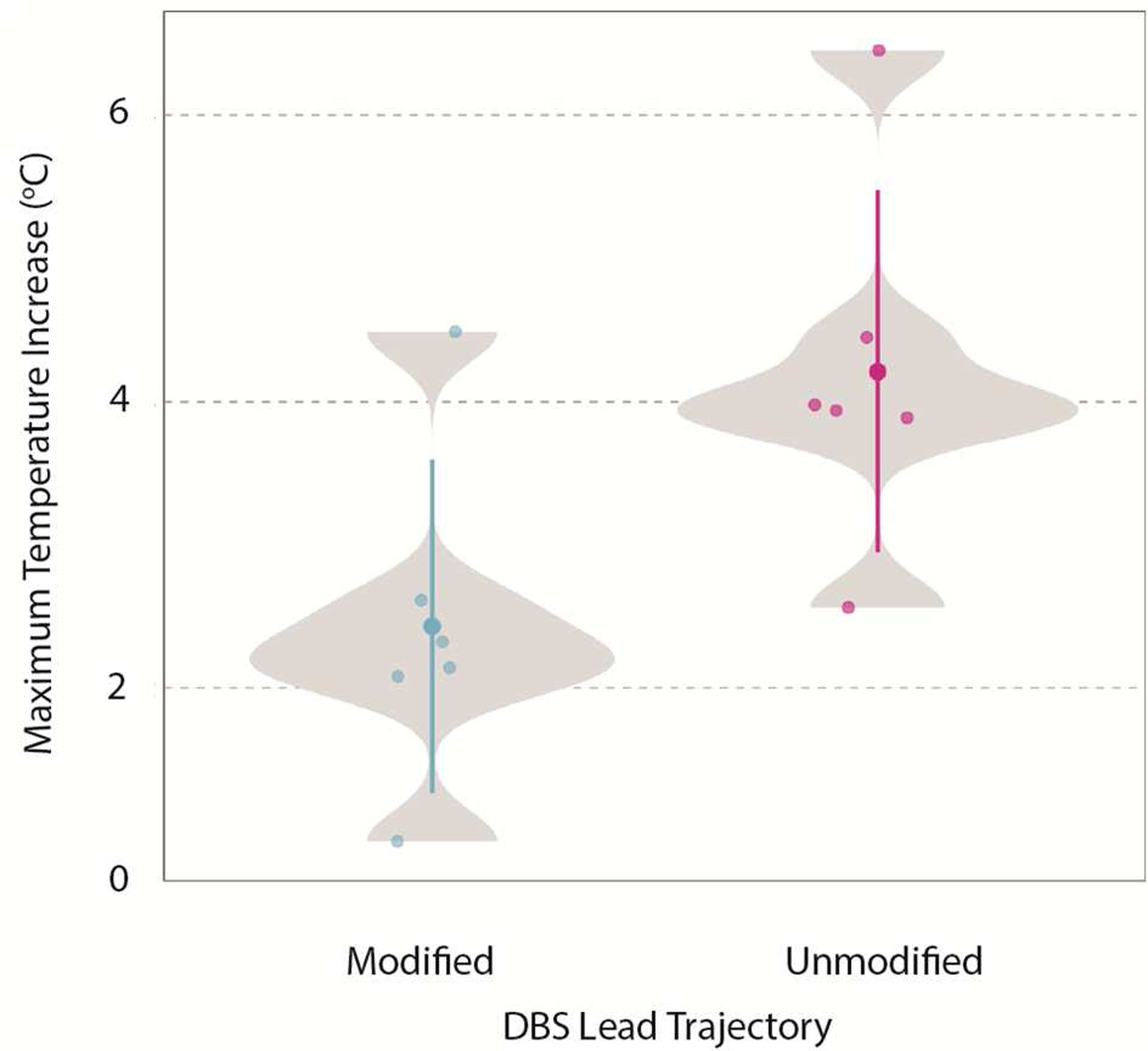
Distribution of ΔT_max_ for the surgically modified and unmodified lead trajectories replicated during phantom experiments.

DBS lead trajectories with 2–3 concentric loops near the surgical burr hole were implemented in six new patients. Subsequently, we replicated these patient-derived trajectories in phantom experiments with the Boston Scientific DBS system. The mean ± standard deviation of ΔT_max_ was 2.43 ± 1.16 °C with a range of 0.93–4.49 °C for the surgically modified trajectories. We then performed experiments with six unmodified lead trajectories previously implemented in other patients by the same neurosurgeons. The mean ± standard deviation of ΔT_max_ was 4.21 ± 1.27 °C with a range of 2.56–6.45 °C for the unmodified trajectories, resulting in almost a 2-fold reduction in RF heating (Fig. 7).

## IV. Discussion and Conclusion

Modern neuroimaging techniques are increasingly needed to inform DBS therapy. While MRI-induced RF heating for patients with DBS systems remains a prominent concern, mitigation efforts have increased in recent years. These contributions include modifying the material and design of DBS leads [24], [25], introducing novel MRI head coil technology to induce a region of low electric field that coincides with the implanted lead’s trajectory on a patient-specific basis [26]–[29] and potential application of ultra-high-field [30] and vertical open-bore scanners which have different orientations of the magnetic and electric fields [31], [32]. However, widespread clinical adoption of these approaches remains limited as they require changes to existing DBS or MRI technology or methodologies.

In this work, we found that placing 2–3 concentric, overlapping loops specifically within 40 mm of the surgical burr hole was most effective for reducing RF-induced heating. For both the Abbott and Boston Scientific DBS systems, the trend of increasing the number of concentric loops—especially near the burr hole—was consistent for both evaluated DBS systems. Notably, manipulating the lead trajectory to low-heating configurations reduced RF heating despite differences in the lead-extension lengths and structures. Our findings were also consistent with the earliest introduction of a DBS lead management prototype that formed 2.25 successive loops ranging from 1.8 to 2.3 cm in diameter near the burr hole [15]. Here, we provide a direct comparison of RF heating across characteristics of the trajectory. By increasing the number of loops from one to two, there was an immediate 3-fold reduction in ΔT_max_. Thus, simple adjustments could be made to create low-heating trajectories.

Our preliminary clinical results demonstrated that modifying the extracranial DBS lead trajectory is feasible within the current surgical workflow at different institutions, without increasing the duration of the surgery as the trajectory loops were created within minutes. The modified DBS lead trajectories reduced RF heating during 3 T MRI by almost 2-folds compared to the unmodified lead trajectories previously implanted by the same neurosurgeons. Surgically modifying the extracranial DBS lead trajectory while focusing on increasing the number of concentric loops and the loops’ placement can effectively mitigate RF heating during 3 T MRI.

Clinical adoption of the trajectory specifications demonstrated great potential and accommodated for different surgical practices across neurosurgeons. Overall, this method can enable safer neuroimaging during MRI at 3 T for patients with DBS systems.

## Clinical Relevance—

Surgically modifying the DBS lead trajectory can be a cost-effective strategy for reducing RF-induced heating during MRI at 3 T.
